# Design Strategy to Create Antibody Mimetics Harbouring Immobilised Complementarity Determining Region Peptides for Practical Use

**DOI:** 10.1038/s41598-020-57713-4

**Published:** 2020-01-21

**Authors:** Tetsuya Kadonosono, Wanaporn Yimchuen, Yumi Ota, Kyra See, Tadaomi Furuta, Tadashi Shiozawa, Maika Kitazawa, Yu Goto, Akash Patil, Takahiro Kuchimaru, Shinae Kizaka-Kondoh

**Affiliations:** 1 School of Life Science and Technology, Tokyo Institute of Technology, Yokohama, 226-8501 Japan; 2 Center for Molecular Medicine, Jichi Medical University, Shimotsuke, 329-0498 Japan

**Keywords:** Peptides, Protein design

## Abstract

Monoclonal antibodies (mAbs) are attractive therapeutics for treating a wide range of human disorders, and bind to the antigen through their complementarity-determining regions (CDRs). Small stable proteins containing structurally retained CDRs are promising alternatives to mAbs. In this report, we present a method to create such proteins, named fluctuation-regulated affinity proteins (FLAPs). Thirteen graft acceptor (GA) sites that efficiently immobilise the grafted peptide structure were initially selected from six small protein scaffolds by computational identification. Five CDR peptides extracted by binding energy calculations from mAbs against breast cancer marker human epithelial growth factor receptor type 2 (HER2) were then grafted to the selected scaffolds. The combination of five CDR peptides and 13 GA sites in six scaffolds revealed that three of the 65 combinations showed specific binding to HER2 with dissociation constants (*K*_D_) of 270–350 nM in biolayer interferometry and 24–65 nM in ELISA. The FLAPs specifically detected HER2-overexpressing cancer cells. Thus, the present strategy is a promising and practical method for developing small antibody mimetics.

## Introduction

Chemically synthesised small antibody mimetics (maximally around 120 aa in peptide length^1^) that specifically bind to the same epitopes as their parental monoclonal antibody (mAb) drugs provide promising options for molecular-targeted therapies. Low-molecular-weight proteins have good tissue penetration, a high excretion rate and low production costs. Thus, in recent years, a variety of small non-immunoglobulin (non-Ig) proteins have been generated via affinity selection of protein libraries that present randomised amino acids on the surface of non-Ig scaffold proteins^2–7^. In parallel, antibody fragmentation technologies have successfully developed small antibody mimetics. However, the current design strategy for reducing the size of the mAb is limited to producing fragment antibodies of 30 kDa in size, which precludes simple production of these fragments by chemical synthesis^8^. Computational protein design that rationally extracts protein fragments from large protein libraries makes it possible to more efficiently create small antigen-binding proteins with a higher affinity toward epitopes^9^.

The antigen-binding surface of mAbs is often composed of six complementarity-determining regions (CDRs), spread across the heavy chain and light chain variable domains. CDR-grafting techniques have been commonly used to humanise exogenous antibodies to reduce their immunogenicity^10^. A typical grafting protocol for antibody humanisation includes the identification of antigen-binding regions in the parental antibody, the selection of a framework region (FR) for providing structural support to the grafted CDRs attached to the human antibody, and, finally, the optimisation of amino acid residues outside of the grafted CDRs to restore or improve the affinity of the humanised antibody^11^. In a similar way, small antibody mimetics targeting lysozyme have been generated by grafting CDRs from a single domain antibody to non-Ig scaffolds, including neocarzinostatin^12^, ubiquitin^13^ and Affitins^14^, thus mimicking the CDR conformations from the parental antibody. These small antibody mimetics have the same CDRs; however, they showed various affinities toward lysozyme (*K*_D_ = 0.5–230 µM), indicating that scaffolds affect the affinity of grafted CDRs. Therefore, when designing antibody mimetics for practical use, it is extremely important both to select appropriate non-Ig scaffolds and to determine the proper peptide sequences in CDRs.

In this report, we aimed to develop a clinically applicable small antibody mimetic termed fluctuation-regulated affinity proteins (FLAPs). The approach for developing FLAPs is based on our recent study that showed the binding activity of target-binding peptides increases by two logs when their structure is immobilised by grafting to a particular site of a fluorescent protein scaffold^15^. Our fast and easy method for developing FLAPs includes computational methods for selecting short peptides from the CDR that help with binding to the target and for determining the graft acceptor (GA) sites, which are crucial for proper immobilisation of the grafted short peptide structure, in small protein scaffolds. Five CDR peptides were extracted from the mAb drugs trastuzumab and pertuzumab that bind human epithelial growth factor receptor type 2 (HER2) and then grafted into 13 selected GA sites of small non-Ig scaffolds. Among the 65 anti-HER2 FLAP candidates created, three bind to the same epitope as the parental mAb, and their *K*_D_ values are within a clinically relevant range that binds to high HER2-expressing cells but reduces on-target off-tumour binding risk, demonstrating that our strategy is a promising method to develop small antibody mimetics that could contribute to molecular-targeted therapy and diagnosis.

## Results

### Strategy for creating FLAPs

**Figure 1:**
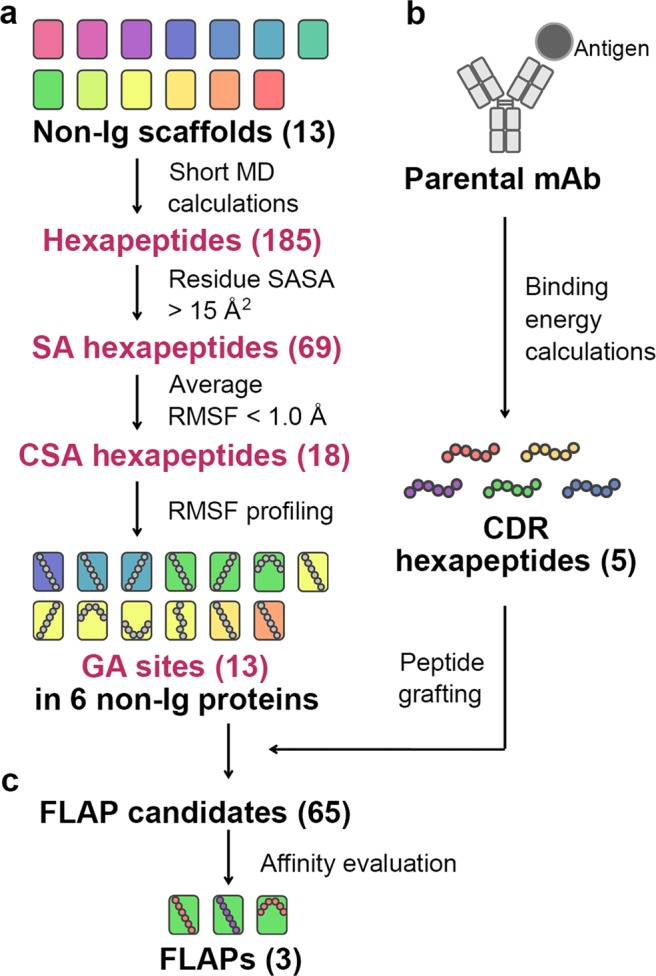
Overview of FLAP design strategy (**a**) Scaffold design. The ideal hexapeptide-grafting sites, GA sites, in the scaffolds were selected based on their structural characteristics and potential to immobilise the grafted hexapeptides as assessed by a computational search. (**b**) Hexapeptide design. Antigen-binding CDR hexapeptides were identified from the parental mAb by the antigen-binding energy calculation and grafted to the GA sites to design FLAPs. (**c**) FLAP design. High-affinity FLAPs were determined by a rapid affinity evaluation approach.

Our previous study revealed that it is crucial for target-binding peptides to be structurally constrained in appropriate scaffolds to ensure that these peptides have high binding affinity^15^. Based on these findings, we aimed to develop a strategy to computationally design small antibody mimetics, named FLAPs, by selecting adequate small protein scaffolds and extracting target-binding sequences from mAb drugs (Fig. 1). Ideal traits of scaffold candidates for drug development include a suitable size (less than 120 amino acids) that allows straightforward chemical synthesis, a low number of disulphide bonds and non-immunogenicity. Taking these traits into consideration, 13 non-Ig proteins of less than 104 amino acids in length that contained fewer than two disulphide bonds as scaffold candidates from endogenous proteins were selected and named Sca1-Sca13, in which the numbering was assigned in order of their amino acid length (Table S1). The longest contiguous sequence of antigen-contact residues in 99% of antibody light chains and 93% of antibody heavy chains is six amino acids (hexapeptide) (Fig. S1). Therefore, hexapeptides were extracted from antibody CDR loops for grafting to the various scaffolds.

### Determination of the criteria for hexapeptide-grafting sites in scaffolds

The structures of the 13 non-Ig scaffolds were equilibrated using molecular dynamics (MD) simulations. The 185 hexapeptides flanked by α-helices, β-strands, or disulphide-bonded cysteine residues in the scaffolds were selected as candidate sites for substitution with CDR hexapeptides, and their solvent accessibility was determined by measuring their buried solvent accessible surface area (SASA) (Fig. S2). The hexapeptides with a SASA value of greater than 15 Å^2^ for each residue were selected as solvent accessible (SA) hexapeptides. As a result, the 185 possible sites were reduced to 69 CDR-grafting candidate sites.

To select ideal grafting sites that enable the grafted hexapeptide to be structurally constrained, the 69 candidate sites were computationally evaluated using our original criteria. The atomic fluctuation of each CDR hexapeptide was evaluated using publicly available structural data from nine single chain Fv (scFv) fragment antibodies and used to determine the appropriate criteria. Since the CDR loop of an antibody is immobilised by FRs, these regions are the best suited for determining appropriate criteria for the immobilised state of peptides. The most flexible hexapeptide sequence (Flex-CDR hexapeptide) in each CDR was determined based on the average root-mean-square fluctuation (RMSF) values of all hydrogen-free atoms calculated using the trajectories of MD simulations (Fig. S3). As a comparison to the highly fluctuating hexapeptides, the RMSF values of linear hexapeptides with the same amino acid sequences as the Flex-CDR hexapeptides were also calculated. These calculations showed that the average RMSF values of the linear hexapeptides were mostly more than 1.5 Å (47/53), whereas the average RMSF values of the hexapeptides in the scFv were mostly less than 1.5 Å (51/53) (Fig. S4). Therefore, the criterion used for identifying structurally constrained hexapeptides was an average RMSF value of less than 1.5 Å.

After applying the above selection criterion, 60 out of 69 sites were found to potentially immobilise a grafted hexapeptide (average RMSF < 1.5 Å), and 18 out of these 60 sites were identified with the potential to highly immobilise a hexapeptide (average RMSF < 1.0 Å). This subset of 18 hexapeptides was named the constrained and solvent accessible (CSA) hexapeptides, and each CSA hexapeptide was named Sca4-1–Sca12-1 to indicate the scaffold in which it is located and its position from the N-terminus (Fig. S5 and Table S2). The 18 CSA hexapeptides, which are potential substitution sequences for antigen binding hexapeptide, within the 7 scaffolds were evaluated further to identify ideal substitution sites.

### Validation of grafting sites that immobilise a grafted hexapeptide

In developing a method to validate the potential of the CSA hexapeptides as ideal hexapeptide-grafting sites, we hypothesised that an ideal hexapeptide-grafting site could immobilise any hexapeptide structure. To test this hypothesis, Flex-CDR hexapeptides in two scFv were replaced with 20 different homo-hexapeptides, and the average RMSF values of the grafted hexapeptides were profiled (RMSF profiling). The homo-hexapeptides included the smallest (Gly-Gly-Gly-Gly-Gly-Gly; G6), bulkiest (Trp-Trp-Trp-Trp-Trp-Trp; W6), most hydrophobic (Ile-Ile-Ile-Ile-Ile-Ile; I6), most acidic (Glu-Glu-Glu-Glu-Glu-Glu; E6) and most basic (Arg-Arg-Arg-Arg-Arg-Arg; R6) peptides among the 6.4 × 10^7^ theoretical hexapeptide species. Therefore, these peptides would be ideal models to validate scaffolds for resistance to all possible factors that may be problematic in immobilising peptides, such as weak atomic forces, repulsion forces and steric hindrances. RMSF profiling revealed that the majority of the average RMSF values of the linear homo-hexapeptides were greater than 2 Å, whereas the average RMSF values of all the homo-hexapeptides grafted into scFv were 1.5 Å or less (Fig. S6). These findings indicate that our method for selecting ideal scaffolds using 20 different homo-hexapeptides is valid.

**Figure 2:**
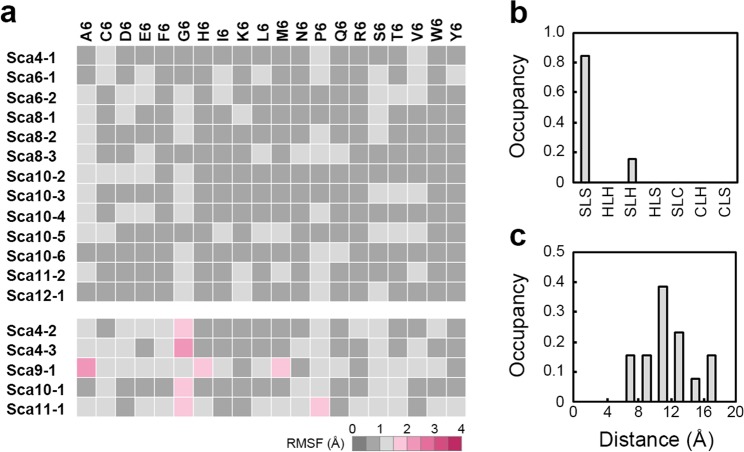
The effect of scaffolds on peptide immobilisation (**a**) RMSF profiling of 18 CSA hexapeptides. Average RMSF values of less or more than 1.5 Å are shown by grey- or magenta-coloured squares, respectively. (**b**) Structural motifs of the flanked loops containing the GA sites. SLS, strand-loop-strand; HLH, helix-loop-helix; SLH, strand-loop-helix; HLS, helix-loop-strand; SLC, strand-loop-cysteine; CLH, cysteine-loop-helix; CLS: cysteine-loop-strand. (**c**) Distance distribution between Cα atoms of the first and sixth amino acids of hexapeptides in the GA sites.

RMSF profiling revealed that 13 out of the 18 CSA hexapeptides in six scaffolds have an average RMSF value of less than 1.5 Å (Fig. 2a, Tables S1 and S2), indicating that these sites have high potential to immobilise the structure of any grafted hexapeptide. The structural features of these 13 CSA hexapeptides, hereinafter referred to as graft acceptor (GA) sites, were then analysed. Interestingly, a half of the scaffolds with GA sites (Sca4, Sca6, Sca8, Sca10, Sca11 and Sca12) have multiple GA sites (Tables S1 and S2). These GA sites were classified according to their adjoining secondary structures. We found that they tended to appear in the strand-loop-strand motifs of scaffolds (Fig. 2b). Next, the influence of peptide deflection on fluctuation was evaluated by analysing the distribution of distances between the Cα atoms of the first and sixth amino acids of GA sites. The results indicate that the distances between the Cα atoms of GA sites are distributed widely, suggesting that the presence of GA sites is independent of the peptide deflection (Fig. 2c).

### Selection of antigen-binding CDR hexapeptides

**Figure 3:**
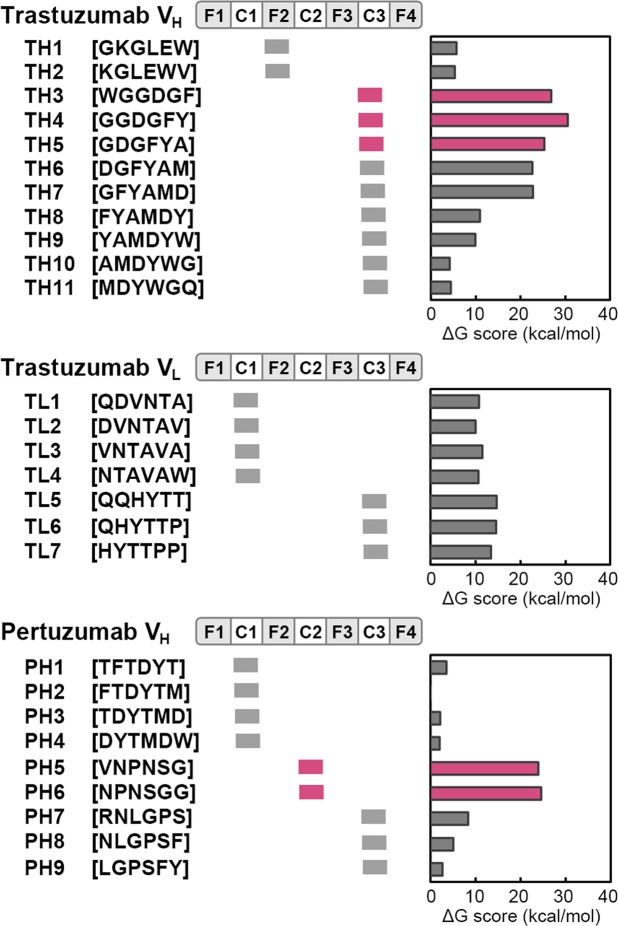
Identification of high-affinity HER2-binding CDR hexapeptides. The names and amino acid sequences of the HER2-binding CDR hexapeptides in the variable domains (V_L_ and V_H_) of trastuzumab and pertuzumab are shown on the left of each panel. F1, C1, F2, C2, F3, C3 and F4 within the upper box in each panel represent the FR1, CDR1, FR2, CDR2, FR3, CDR3 and FR4 domains of the indicated antibodies, respectively. The position of each hexapeptide is shown under the box. The binding energy loss caused by the replacement of each HER2-binding CDR hexapeptide with an alanine homo-hexapeptide is shown in the right graphs. The high-affinity HER2-binding CDR hexapeptides are indicated by magenta colouring.

The results thus far suggested that the structure of the hexapeptide from a mAb CDR can be immobilised when it is grafted at the GA sites in scaffolds. We chose HER2 as a model target for creating FLAPs using our strategy, since this molecule is one of the most important target molecules of breast cancer and, thus, there is an abundance of data describing mAbs against HER2^16^. The eleven (TH1–TH11), seven (TL1–TL7) and nine (PH1–PH9) CDR hexapeptides that may bind to HER2 were selected from the V_H_ domain of trastuzumab, V_L_ domain of trastuzumab and V_H_ domain of pertuzumab, respectively, based on the antigen-contact residues in crystal structures (Fig. 3). These hexapeptides were replaced *in silico* with an alanine homo-hexapeptide to calculate the binding energy loss caused by the replacement. The V_L_ domain of pertuzumab had no HER2-binding CDR hexapeptide. Three (TH3, TH4, TH5) and two (PH5, PH6) CDR hexapeptides from the candidate hexapeptides in trastuzumab and pertuzumab, respectively, showed drastically reduced binding energy following their replacement (Fig. 3). The results suggest that these sequences are crucial for maintaining strong interactions with HER2.

These five CDR hexapeptides were each grafted into the previously selected 13 GA sites in six scaffolds to generate a total of 65 FLAP candidates: For convenience, the FLAP candidates were named as [Name of GA site]-[Name of grafted CDR hexapeptide] (Sca4-1-TH3–Sca12-1-PH6). As expected, the RMSF values of all grafted CDR hexapeptides were less than 1.5 Å (Fig. S7a), confirming that our structural constraint index (1.5 Å) and screening methods are reliable. Since the structure of the immobilised peptide varies depending on the scaffold structure, the root-mean-square deviation (RMSD) values of the CDR hexapeptides in the scaffolds varied from those in their original crystal structures (Fig. S7b).

### Rapid identification of antigen-binding FLAPs

**Figure 4:**
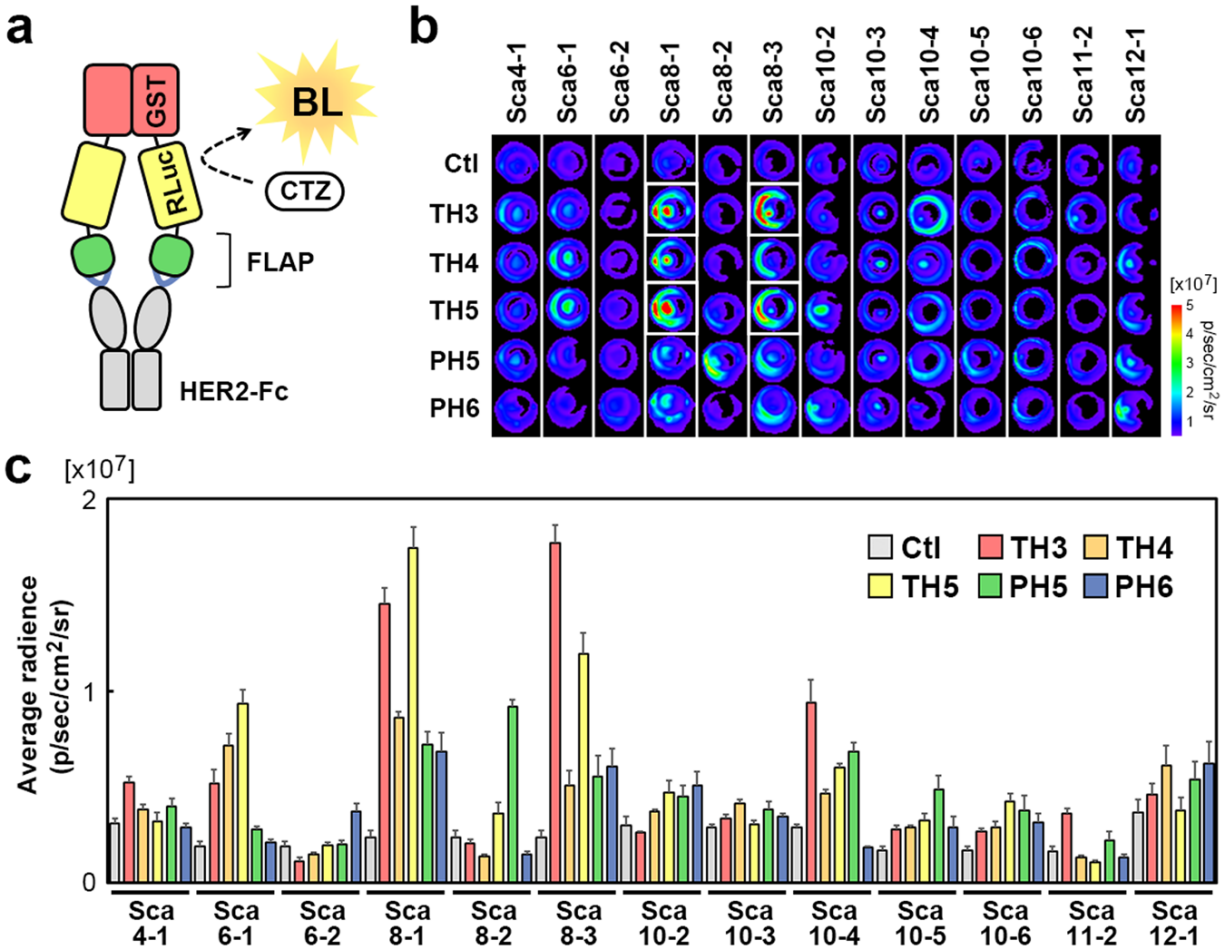
Rapid evaluation of FLAP candidate**s**. (**a**) Schematic diagram of the RLuc-fused FLAP structure and the principal for affinity determination by BLI-ELISA. The binding affinity of FLAP candidates to HER2-Fc was quantified by the bioluminescence (BL) signal intensity obtained by the reaction between RLuc and coelenterazine (CTZ). (**b**,**c**) The HER2-Fc binding activity of the 65 FLAP candidates. FLAP candidates, each consisting of a scaffold (any of Sca4-1 to Sca12-1) and CDR-derived hexapeptide (any one of TH3, TH4, TH5, PH5, or PH6), were evaluated by BLI-ELISA for binding activity to HER2-Fc. Control (Ctl) is the specified RLuc-fused scaffold without a CDR-derived hexapeptide. A representative BL image of the BLI-ELISA plates is shown (**b**), and the average radiance of each well from three independent experiments is shown as the mean ± SEM (**c)**. White frames in (**b**) indicate the wells of Sca8-1-TH3, Sca8-1-TH5, Sca8-3-TH3 and Sca8-3-TH5.

The FLAPs with high binding affinity for HER2 were experimentally identified by screening with a fast and easy method using bioluminescence, which is a highly sensitive method to quantitatively analyse target-binding proteins even without protein purification^17^. The FLAP candidates fused with glutathione-S-transferase (GST) and *Renilla* luciferase 8.6–535 (RLuc)^18^ were expressed as a dimeric form in *Escherichia coli*, and the resulting crude extract was used to examine the binding activities of FLAP candidates to HER2 using a bioluminescence imaging (BLI)-ELISA (Fig. 4a). Among the 65 FLAP candidates, strong bioluminescence signals were detected in all the FLAP candidates with Sca8-1 and Sca8-3 GA sites (Fig. 4b). In particular, Sca8-1-TH3, Sca8-1-TH5, Sca8-3-TH3 and Sca8-3-TH5 showed very strong bioluminescence signals (Fig. 4b,c).

The HER2-binding peptide (*K*_D_ = 536 nM) were reported to detect HER2 high-expression tumours *in vivo*^19^, suggesting that a moderate affinity is clinically relevant. All candidates showed *K*_D_ values between 270 and 550 nM when the FLAP structure of each of these four candidates was purified as a monomeric form and their binding affinity to HER2 was determined by biolayer interferometry (Fig. S8 and Table 1). These values are about 570–1200 times higher than the *K*_D_ value of the parental trastuzumab-Fab (Table 1). In addition, the affinity of these FLAPs in ELISA is 24–65 nM, about 350–960 times higher than that of the parental trastuzumab-Fab, but 150–420 times lower than the *K*_D_ values of the peptides fused to the C-terminus of the scaffolds (Sca8-Cterm-TH3 and Sca8-Cterm-TH5), consistent with the previous observations^15^ (Fig. S9 and Table 1).

**Table 1:** Affinity toward HER2 of trastuzumab, the Sca8 scaffold and Sca8-based FLAPs.

Trastuzumab, scaffold, and FLAPs	Biolayer interferometry		ELISA	
	*K*_D_ (nM)	*k*_on_ (10^4^ M^–1^s^–1^)	*k*_off_ (10^–2^s^–1^)	*K*_D_ (nM)
Trastuzumab (divalent)	ND^a^	ND	ND	0.015 ± 0.0032
Trastuzumab-Fab	0.47	290 ± 30	0.14 ± 0.015	0.068 ± 0.014
Sca8-Ctl	ND	ND	ND	>10000
Sca8-Cterm-TH3	ND	ND	ND	>10000
Sca8-Cterm-TH5	ND	ND	ND	>10000
Sca8-1-TH3	350	2.6 ± 0.12	0.90 ± 0.077	64 ± 10
Sca8-1-TH5	270	3.9 ± 0.29	1.1 ± 0.27	24 ± 3.2
Sca8-3-TH3	330	2.7 ± 0.30	0.87 ± 0.14	65 ± 8.1
Sca8-3-TH5	550	2.6 ± 0.49	1.4 ± 0.022	52 ± 14
Sca8-2-TH5	ND	ND	ND	860 ± 45

^a^ND, Not determined.

### Characterisation of the anti-HER2 FLAPs

**Figure 5:**
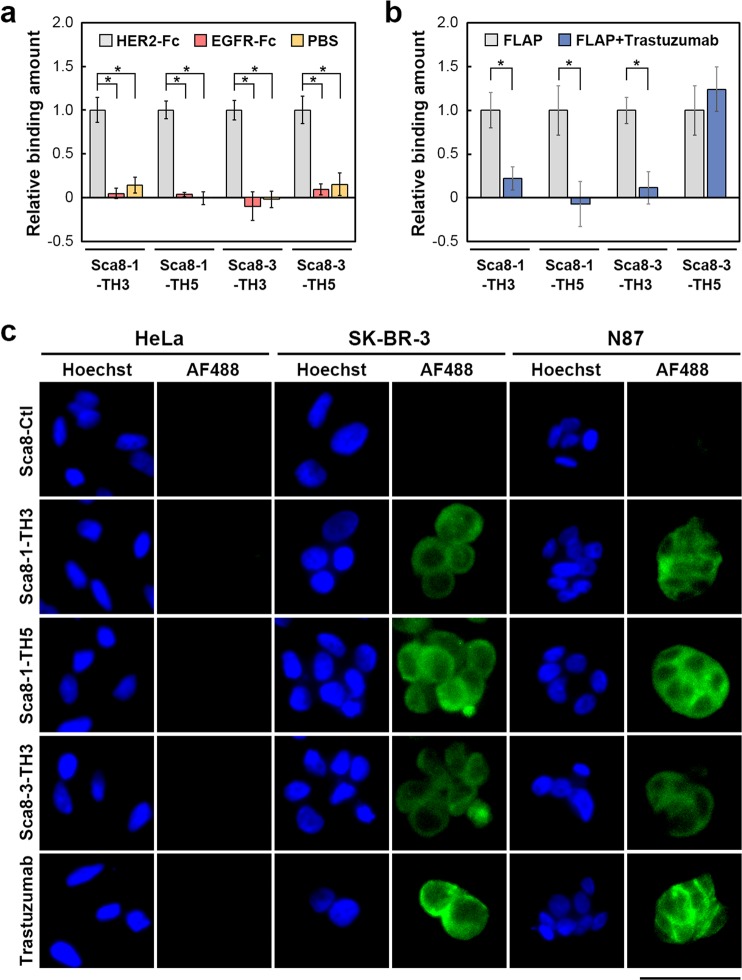
Characterisation of anti-HER2 FLAPs. (**a**) Specific binding of anti-HER2 FLAP candidates to HER2. FLAP candidates binding to HER2-Fc, EGFR-Fc, or non-coated well (PBS) were evaluated by ELISA. The mean ± SEM of three independent experiments is shown; *p < 0.05. (**b**) Binding competition between anti-HER2 FLAP candidates and their parental mAb. His-tagged FLAPs (100 nM) were mixed with 100 nM trastuzumab or buffer, and FLAP candidates binding to HER2-Fc were evaluated by ELISA. The mean ± SEM of three independent experiments is shown; *p < 0.05. (**c**) Immunostaining of HeLa, SK-BR-3/Luc, or N87 cells with His-tagged FLAPs or trastuzumab (green) and Hoechst (blue). Scale bar = 50 µm.

The four anti-HER2 FLAP candidates were examined for their binding specificity as alternatives to the primary antibody. When the affinities of these FLAPs to HER2-Fc, epidermal growth factor receptor (EGFR)-Fc, or non-coated wells were evaluated by ELISA, the FLAPs specifically bound to HER2-Fc and hardly bound to EGFR-Fc and non-coated wells (Fig. 5a). As they all share their target binding sequences with trastuzumab, competitive binding was examined by incubating these FLAPs with HER2-Fc proteins in the presence of trastuzumab. The binding amount of three FLAPs, Sca8-1-TH3, Sca8-1-TH5, and Sca8-3-TH3, decreased significantly in the presence of trastuzumab (Fig. 5b), confirming that the FLAPs bind to the same epitope as trastuzumab. In contrast, Sca8-3-TH5 was suggested to bind to HER2-Fc with the different epitope from trastuzumab (Fig. 5a,b). Therefore, we hereinafter regard these three candidates, Sca8-1-TH3, Sca8-1-TH5, and Sca8-3-TH3, as anti-HER2 FLAPs. When HeLa cells (hardly express HER2), SK-BR-3/Luc cells (express HER2), or N87 cells (express HER2) were incubated with the FLAPs followed by incubation with a corresponding fluorescence-labelled secondary antibody, strong fluorescence was observed in the SK-BR-3/Luc and N87 cells but not in the HeLa cells (Fig. 5c). These results indicate that the anti-HER2 FLAPs have high specificity toward HER2.

## Discussion

In this study, we clearly demonstrated that our strategy of grafting constrained CDR peptides into appropriate scaffolds selected by using our original criteria is a reliable method for generating high-performance antibody mimetics that bind to the same epitopes as the mAbs from which the CDR peptides were derived. The proposed design strategy successfully developed a constrained CDR-grafted non-Ig affinity protein, FLAP (Figs. 1–3), which demonstrated efficient binding to HER2 (Figs. 4 and 5b and Table 1), and usability for detecting antigen-expressing cells (Fig. 5c). In addition, the FLAPs were found to barely bind to EGFR, which belongs to the same receptor family as HER2, indicating that the mAb specificity of the target-binding peptides is also retained in the FLAPs (Fig. 5a). The FLAP properties of a sufficiently small molecular size to be amenable for chemical synthesis and an epitope identical to that of the parental antibody are significant advantages of FLAPs over fragment antibodies, which are relatively large molecules. Our design strategy for developing FLAPs can be applied to many mAbs, facilitating the development of alternatives to antibodies already used in clinical and basic research.

Amino acid sequences of antibody CDRs seem to be optimized during the affinity maturation process to decrease the entropic cost on antigen binding and to increase the enthalpic contribution^20–23^. Our computational prediction provides specific indicators for structural immobilisation. It also revealed that almost all CDRs are immobilised in antibody FRs (Fig. S4) and that FRs retain a high capacity to reduce the fluctuation of various peptides (Fig. S6), clearly showing that FRs function as a general scaffold to reduce the entropic energy loss of CDRs on antigen binding. Furthermore, CDR-derived short peptides often lose their antigen-affinity when released from FRs. Ideally, the CDR-derived short peptides in FLAPs would be expected to have high affinity, similar to that of the parental antibodies, because they are immobilised in a manner like they are in antibody FRs. However, our method developed only three FLAPs out of 65 anti-HER2 FLAP candidates, indicating that the CDR peptides in most of FLAP candidates do not have optimally immobilised structures. The anti-HER2 FLAP candidates were immobilised (Fig. S7a) with various structures (Fig. S7b) and various affinities against HER2 (Fig. 4 and Table 1), confirming that an appropriate CDR hexapeptide structure is also required for enthalpic interaction. Further optimisation of the immobilised structure of pertuzumab-derived CDR hexapeptides in more suitable scaffolds may facilitate the development of high-affinity FLAPs with these hexapeptides. Although a highly accurate structure prediction is needed to validate the accurate enthalpy and entropy changes on antigen binding, the currently applied computational calculation has several limitations, such as the lack of a suitable general algorithm and its high computational complexity. Thus, the semi-rational strategy presented here is one of the most feasible methods for efficiently developing antibody mimetics.

We successfully designed anti-HER2 FLAPs with our new method but the affinities of these FLAPs were still lower than those of the parent antibodies. A possible engineering strategy to increase the affinity of FLAPs involves multiple grafting of constrained CDR-derived peptides, which may be successful because antibody paratopes are often formed by a combination of dominant CDRs and ancillary CDRs. In the case of trastuzumab, for example, CDR-H3 is the dominant CDR, and CDR-L1 and CDR-L3 are the ancillary CDRs (Fig. 3), but the designed FLAPs in this research each had only one CDR hexapeptide from CDR-H3. The additional grafting of CDR hexapeptides from CDR-L1 and CDR-L3 onto the FLAPs with favourable geometry would likely improve the affinity of these FLAPs. Scaffolds such as Sca10 have multiple geometrically proximal GA sites (Table S2) and thus may be suitable for grafting multiple CDR-derived peptides to yield higher affinity FLAPs and biparatopic FLAPs.

Notably, HER2 is expressed at low levels in a wide variety of vitally important normal tissues and thus there is a concern about side effects in HER2-targeting therapies using mAbs^24^. Base on the recent study using a bivalent anti-HER2 engineered antibody^25^, the moderate affinity (*K*_D_) of FLAPs created in this study is within a clinically relevant range, suggesting that mono and multivalent anti-HER2 FLAPs are promising options for developing specific drugs against HER2-amplified cancers. The HER2-binding affibodies or peptides were reported to detect HER2 high-expression tumours *in vivo*^19,26–28^. Their affinity (*K*_D_) toward recombinant HER2 proteins were the range of 22 pM–536 nM, indicating that FLAPs have enough affinity for the development of tumour imaging probes.

Sca8-3-TH5 showed similar affinity and specificity to HER2 with three FLAPs (Table 1, Fig. 5a) and can be used for the detection of HER2-expressing SK-BR-3 and N87 cells (Figs. 5c and S10), but its epitope seemed to be different from trastuzumab (Fig. 5b), suggesting that novel epitope-binding surface was constructed by the grafting of the CDR hexapeptide, TH5. Although further analysis is required to clarify binding mode of Sca8-3-TH5, this unexpected result would indicate the usefulness of our method to develop a practical antibody mimetics with different paratope from parental mAb.

We initially selected 13 non-Ig human protein scaffold candidates that had sizes amenable to chemical synthesis and had a low number of disulphide bonds, and then using our own validation method six suitable proteins were identified. Among them, two are well-studied non-Ig scaffold proteins, the SH3 domain of Fyn (Sca4, fynomer) and the fibronectin type III domain (Sca8, FN3 or adnectin) (Fig. 2a, Tables S1 and S2), suggesting that our strategy is a very effective method for screening practical non-Ig scaffold proteins. These two domains have relatively high melting transitions (*T*_m_ = 72 °C for fynomer and 84 °C for FN3), low immunogenicity and their biodistribution can be improved by Fc-fusion and PEGylation techniques. Thus, many mutant proteins have been developed using these domains as scaffolds, and some are under clinical evaluations for the treatment of cancer, hypercholesterolemia, cachexia and plaque psoriasis^29,30^. As FLAPs with these scaffolds are suitable for clinical application, they will be used in future research to advance to FLAPs development for clinical application.

Characterisation of the other four scaffolds (Sca6, ras-binding domain of RalGEF; Sca10, beta 2-microglobulin; Sca11, growth factor receptor-bound protein 7; Sca12, constant region of IgG-light chain (CL)) for clinical applications has received less attention. Only thermal stability is known for Sca10 (*T*_m_ = 60 °C) and Sca12 (*T*_m_ = 53 °C for murine CL domain)^31^. Although future studies need to verify whether these scaffolds are suitable for clinical application, we have shown that they are potential candidates that can be used as non-Ig scaffolds.

Our study demonstrates the general concept for designing high-performance antibody mimetics by grafting constrained antigen-binding peptides into non-Ig scaffolds. This strategy is applicable to the generation of antibody mimetics and other functional proteins, including receptor ligands, cytokines, inhibitors and enzymes. Therefore, this study may open new avenues for developing novel technology platforms in protein engineering and biopharmaceutical design.

## Methods

### MD simulations

The initial coordinates of nine scFv molecules and 13 scaffolds were taken from PDB accession codes 2YC1, 3JUY, 3UX9, 4BUH, 4KV5, 4UT7, 4X4X, 5C2B and 5D9Q, and 4HSV, 4ZAI, 2QKQ, 3UA7, 2YUU, 2RGF, 3TSV, 1TTG, 3FIA, 4EN3, 1WGR, 3PGF and 4DZ8, respectively. The structures of linear CDR peptides, model linear peptides, model hexapeptide-grafting scFv molecules, model hexapeptide-grafting scaffold candidates and CDR peptide-grafting scaffolds were generated using Discovery studio 3.1 (Accelrys, San Diego, CA, USA). The systems were optimised via energy minimisation and equilibrated with backbone restraints. Production runs were performed for at least 10 ns for trajectory analysis. All MD simulations were performed using the Amber 14 and 16 program packages^32^ on TSUBAME (Global Scientific Information and Computing Center, Tokyo Institute of Technology). The Amber ff14SB force field and the GB/SA implicit solvent model were used. The time-step for MD simulations was set to 2 fs with the SHAKE algorithm. A nonbonded cutoff of 999.9 Å was used. The temperature was kept constant at 300 K using the Berendsen rescaling method.

The RMSFs during the final 5 ns of each production run were calculated to investigate the backbone fluctuations in each system using the cpptraj module. The SASAs of the final structures of each production run were calculated to identify the solvent-accessible residues in the 13 scaffolds using the cpptraj module.

### Identification of GA sites

The GA sites were identified via an *in silico* two-step method. Firstly, the CSA hexapeptides were selected based on particular characteristics. The following filters were applied to narrow down the potential hexapeptides:

1. The hexapeptides were from a loop region sequentially flanked by α-helices, β-strands, or disulphide-bonded cysteine residues.
2. The buried SASA of each residue was larger than 15 Å^2^.
3. The average RMSF of the hexapeptides was less than 1.0 Å.

Next, each CSA hexapeptide in the scaffolds was computationally replaced with 20 different homo-hexapeptides, including Ala-Ala-Ala-Ala-Ala-Ala (A6), Cys-Cys-Cys-Cys-Cys-Cys (C6), Asp-Asp-Asp-Asp-Asp-Asp (D6), Glu-Glu-Glu-Glu-Glu-Glu (E6), Phe-Phe-Phe-Phe-Phe-Phe (F6), Gly-Gly-Gly-Gly-Gly-Gly (G6), His-His-His-His-His-His (H6), Ile-Ile-Ile-Ile-Ile-Ile (I6), Lys-Lys-Lys-Lys-Lys-Lys (K6), Leu-Leu-Leu-Leu-Leu-Leu (L6), Met-Met-Met-Met-Met-Met (M6), Asn-Asn-Asn-Asn-Asn-Asn (N6), Pro-Pro-Pro-Pro-Pro-Pro (P6), Gln-Gln-Gln-Gln-Gln-Gln (Q6), Arg-Arg-Arg-Arg-Arg-Arg (R6), Ser-Ser-Ser-Ser-Ser-Ser (S6), Thr-Thr-Thr-Thr-Thr-Thr (T6), Val-Val-Val-Val-Val-Val (V6), Trp-Trp-Trp-Trp-Trp-Trp (W6) and Tyr-Tyr-Tyr-Tyr-Tyr-Tyr (Y6), after which MD simulations of each structure was calculated. CSA hexapeptides in which the average RMSF of all replaced homo-hexapeptides was less than 1.5 Å were identified as GA sites.

### Identification of antigen-binding CDR hexapeptides

Antigen-binding CDR hexapeptides of mAbs were identified using the *in silico* alanine hexapeptide scanning method. The binding energies of trastuzumab and pertuzumab toward HER2 in their complex structures (PDB accession codes 1N8Z and 1S78, respectively) were predicted by calculating the total energy difference after energy minimisation and equilibration using the Amber ff14SB force field between bound and unbound structures, referred to as ΔG scores. Each CDR-derived hexapeptide sequence was computationally mutated to an alanine hexapeptide, and the top three and two sequences of trastuzumab and pertuzumab, respectively, with ΔG scores that decreased by at least 23 kcal/mol after alanine hexapeptide mutation were selected.

### Grafting of antigen-binding CDR hexapeptides onto scaffolds

The antigen-binding CDR hexapeptides were computationally grafted into scaffolds to generate FLAP candidates by replacing the residues of GA sites in the scaffolds with corresponding residues of the CDR hexapeptides. Structures were optimized by MD simulations of each FLAP candidate. The heavy-atom RMSD of the grafted CDR hexapeptides between the peptides in the antibody CDR and those in the FLAP candidates was calculated from crystal structures of the antibodies and predicted structures of the FLAP candidates using the cpptraj module.

### Plasmid construction and expression of FLAP candidates

The cDNA encoding fusion proteins composed of a His-tag, RLuc, GGGS linker and scaffolds (in this order) used for BLI-ELISAs or the cDNA encoding His-tagged scaffolds used for ELISA were inserted into the multi-cloning site of the pGEX-6P-3 plasmid (GE Healthcare, Little Chalfont, UK) by the Gibson assembly technique (New England Biolabs, Ipswich, MA, USA). The cDNA encoding FLAP candidates were constructed by site-directed mutation of scaffold sequences. These plasmids were introduced into BL21 (DE3) pLys S cells (Promega, Fitchburg, WI, USA), after which the GST-tagged fusion proteins were expressed as described previously^15^.

### BLI-ELISA

Bacteria expressing each RLuc-fused FLAP candidate were lysed by freeze-thaw treatment and sonication. After ultracentrifugation at 210,000 × *g* for 15 min, bioluminescence imaging of the supernatant was acquired for 30 s to determine the concentration of sample proteins based on the sample reaction with the RLuc substrate coelenterazine-h (10 ng/µl in PBS; Promega) using an IVIS Lumina *in vivo* imaging system (PerkinElmer, Waltham, MA, USA). Each BLI-ELISA was carried out at room temperature in 96-well black plates. The wells were first coated with HER2-Fc (50 ng/50 µl in PBS; ACRO Biosystems, Newark, DE, USA) overnight, blocked with PBS containing 2% Perfect-Block (MoBiTec, Göttingen, Germany) (PBS-PB) for 2 h, incubated with 100 µl of 1 µM sample proteins for 1 h, and washed three times with PBS containing 0.05% Tween-20 (PBS-T). After the addition of coelenterazine-h to each well, bioluminescence imaging was acquired for 30 s.

### ELISA

GST-fused and His-tagged FLAPs were purified from the supernatants of bacterial extracts by affinity chromatography using GSTrap HP columns (GE Healthcare) with an AKTA pure 25 system (GE Healthcare). After protease-cleavage by PreScission Protease (GE Healthcare), His-tagged monomeric FLAPs were purified using HisTrap HP columns (GE Healthcare) with an AKTA pure 25 system and dialysed against PBS. Each ELISA was carried out at room temperature in 96-well black plates. The wells were first coated with HER2-Fc (50 ng/50 µl in PBS) overnight, blocked with 2% PBS-PB for 2 h, incubated with sample proteins in 2% PBS-PB for 1 h, and then washed three times with 0.05% PBS-T. After incubation with 1000-fold diluted HRP-conjugated anti-His-tag antibodies (Abcam, Cambridge, MA, USA) in 2% PBS-PB for 1 h, the wells were washed three times with 0.05% PBS-T and three times with PBS. Fluorescence signals generated by using a QuantaRed Enhanced Chemifluorescent HRP Substrate kit (Thermo Fisher Scientific) were measured by using an Infinite F500 (Tecan, Mannedorf, Switzerland) with specific filters (Ex/Em = 535 nm/590 nm). For the detection of trastuzumab (Chugai Pharmaceutical, Tokyo, Japan) and trastuzumab-Fab (provided by Sysmex Corporation, Kobe, Japan), HRP-conjugated anti-human Kappa light chain (Abcam) and HRP-conjugated anti-human IgG (Fab) (Merck KGaA, Darmstadt, Germany), respectively, were used.

For specificity evaluation, the wells were incubated overnight with HER2-Fc (50 ng/50 µl in PBS), EGFR-Fc (50 ng/50 µl in PBS; R&D systems, MN, USA), or 50 µl PBS, and then used as the antigen-coated wells in ELISAs.

For binding competition assays, 100 nM His-tagged monomeric FLAPs mixed with 100 nM trastuzumab in 2% PBS-PB were used as the sample protein in ELISAs.

### Biolayer interferometry

The kinetics of FLAP binding to HER2 was studied using a FortéBio Octet Red instrument. The assays were performed at 30 °C in 96-well black plates. HER2-Fc was biotinylated using a biotin labelling kit (Dojindo, Kumamoto, Japan), and 100 nM biotinylated HER2-Fc in the kinetic buffer (0.1% BSA, 0.002% Tween-20 in PBS) was used to load the ligand onto the surface of streptavidin biosensors for 300 s. After washing (30 s) and equilibrating (60 s) the biosensor, the association of the ligand on the biosensor to the analyte in solution (100–1000 nM His-tagged monomeric FLAPs or 1.25–20 nM trastuzumab-Fab) was measured for 300 s. The dissociation of the interaction was subsequently measured for 300 s. Systematic baseline drift correction was done by subtracting the shift recorded for sensors loaded with ligand but incubated without analyte. Data analysis and curve fitting were done using Octet software version 11.0. Experimental data were fitted with the binding equations available for a 1:1 interaction with local fitting and the mean ± standard error of the mean (SEM) values of *k*_on_ and *k*_off_ were calculated from the data of five different concentrations of an analyte. The *K*_D_ was calculated as the ratio of *k*_off_/*k*_on_.

### Cell immunostaining

The human cervical cancer cell line HeLa and human gastric cancer cell line N87 or human breast cancer cells expressing firefly luciferase SK-BR-3/Luc were obtained from the RIKEN Bio-Resource Center (Tsukuba, Japan) or JCRB Cell Bank (Osaka, Japan), respectively, and maintained at 37 °C in 5% FBS-DMEM (Nacalai Tesque, Kyoto, Japan) supplemented with penicillin (100 U/ml) and streptomycin (100 µg/ml) (Nacalai Tesque). The cells (1.0 × 10^4^ cells/well) were seeded on a slide chamber plate, cultured for 16 h and fixed by treatment with a 4% paraformaldehyde solution (Nacalai Tesque) for 10 min at 25 °C. The cells were blocked with 1% BSA/PBS for 1 h at 25 °C. After the addition of 50 nM purified His-tagged FLAPs or 5 nM trastuzumab in 1% BSA/PBS to the chamber, the cells were incubated for 16 h at 4 °C. Thousand-fold diluted Alexa Fluor 488-conjugated mouse anti-His tag secondary antibody (Medical & Biological Laboratories, Aichi, Japan) or Alexa Fluor 488-conjugated mouse anti-human IgG Fc secondary antibody in 1% BSA/PBS were used to fluorescently label the His-tagged FLAPs or trastuzumab, respectively. After washing three times with PBS, the stained cells were mounted with Fluoromount (Diagnostic ByoSystems, CA, USA) containing 1/1000 Hoechst 33342 (Nacalai Tesque). All photos were taken using a BZ-X700 microscope (Keyence, Osaka, Japan).

### Statistical analysis

Data are presented as means ± SEM and were statistically analysed with a two-sided Student’s *t*-test; *p* values of < 0.05 were considered statistically significant.

## Supplementary information

Supplementary Information.
