# Supplementary material for: Design Strategy to Create Antibody Mimetics Harbouring Immobilised Complementarity Determining Region Peptides for Practical Use
Source: Sci Rep. 2020 Jan 21;10:891. doi: 10.1038/s41598-020-57713-4 (PMC6972867; doi:10.1038/s41598-020-57713-4)
Supplement: Supplementary file 1 — Supplementary Information. [file 41598_2020_57713_MOESM1_ESM.docx]

**Supplementary Information for**

**Title**

Design Strategy to Create Antibody Mimetics Harbouring Immobilised Complementarity Determining Region Peptides for Practical Use

**Authors**

Tetsuya Kadonosono, Wanaporn Yimchuen, Yumi Ota, Kyra See, Tadaomi Furuta, Tadashi Shiozawa, Maika Kitazawa, Yu Goto, Akash Patil, Takahiro Kuchimaru, and Shinae Kizaka-Kondoh

**This PDF file includes:**

Tables S1 to S2

Figs. S1 to S10

**Table S1. List of scaffold candidates**

| Scaffold candidate | Protein/domain/peptide name | Structural annotation | PDB ID | Region | Length | No. of SS bond |
| --- | --- | --- | --- | --- | --- | --- |
| Sca1 | CXCL4L1 | Small cytokines | 4HSV | 9−54 | 46 | 2 |
| Sca2 | CXCL13 | Small cytokines | 4ZAI | 10−55 | 46 | 2 |
| Sca3 | SAM domain of ephrin type-B receptor 4 | Orthogonal bundle | 2QKQ | 908−968 | 61 | 0 |
| Sca4^a^ | SH3 domain of Fyn | Others | 3UA7 | 81−143 | 63 | 0 |
| Sca5 | C1 domain of protein kinase C | Others | 2YUU | 1−83 | 83 | 0 |
| Sca6^a^ | Ras-binding domain of RalGEF | Roll | 2RGF | 11−97 | 87 | 0 |
| Sca7 | PDZ domain of ZO-1 MAGUK | Roll | 3TSV | 420−512 | 93 | 0 |
| Sca8^a^ | Fibronectin type III domain | Immunoglobulin-like | 1TTG | 1−94 | 94 | 0 |
| Sca9 | EH1 domain of intersectin-1 | Orthogonal bundle | 3FIA | 6−103 | 98 | 0 |
| Sca10^a^ | β2-Microglobulin | Immunoglobulin-like | 4EN3 | 1−99 | 99 | 1 |
| Sca11^a^ | Growth factor receptor-bound protein 7 | Roll | 1WGR | 1−100 | 100 | 0 |
| Sca12^a^ | Constant region of IgG-light chain (CL) | Immunoglobulin-like | 3PGF | 111−213 | 103 | 0 |
| Sca13 | Constant region of IgG-heavy chain (CH2) | Immunoglobulin-like | 4DZ8 | 238−341 | 104 | 1 |

^a^ These scaffolds have GA sites.

**Table S2. Average root-mean-square fluctuation (RMSF) and solvent accessible surface area (SASA) of constrained and solvent accessible (CSA) hexapeptides**

| CSA hexapeptide [region] | Average RMSF (Å) | SASA (Å^2^) | | | | | |
| --- | --- | --- | --- | --- | --- | --- | --- |
|  |  | 1 | 2 | 3 | 4 | 5 | 6 |
| Sca4-1 [90−95]^a^ | 0.76 | 109 | 116 | 44 | 22 | 86 | 57 |
| Sca4-2 [91−96] | 0.83 | 116 | 44 | 22 | 86 | 57 | 156 |
| Sca4-3 [92−97] | 0.93 | 44 | 22 | 86 | 57 | 156 | 69 |
| Sca6-1 [74−79]^a^ | 0.84 | 56 | 92 | 124 | 22 | 54 | 97 |
| Sca6-2 [75−80]^a^ | 0.83 | 92 | 124 | 22 | 54 | 97 | 15 |
| Sca8-1 [50−55]^a^ | 0.93 | 28 | 96 | 19 | 88 | 36 | 59 |
| Sca8-2 [51−56]^a^ | 0.91 | 96 | 19 | 88 | 36 | 59 | 53 |
| Sca8-3 [63−68]^a^ | 0.95 | 42 | 101 | 47 | 47 | 44 | 16 |
| Sca9-1 [67−72] | 0.79 | 126 | 112 | 115 | 108 | 15 | 39 |
| Sca10-1 [42−47] | 0.63 | 137 | 64 | 115 | 127 | 39 | 150 |
| Sca10-2 [43−48]^a^ | 0.62 | 64 | 115 | 127 | 39 | 150 | 41 |
| Sca10-3 [44−49]^a^ | 0.58 | 115 | 127 | 39 | 150 | 41 | 39 |
| Sca10-4 [45−50]^a^ | 0.57 | 127 | 39 | 150 | 41 | 39 | 98 |
| Sca10-5 [73−78]^a^ | 0.66 | 30 | 99 | 94 | 50 | 56 | 24 |
| Sca10-6 [85−90]^a^ | 0.74 | 97 | 53 | 49 | 120 | 141 | 51 |
| Sca11-1 [17−22] | 1.00 | 120 | 89 | 50 | 33 | 55 | 95 |
| Sca11-2 [18−23]^a^ | 0.99 | 89 | 50 | 33 | 55 | 95 | 87 |
| Sca12-1 [151−156]^a^ | 0.80 | 40 | 154 | 39 | 106 | 63 | 18 |

^a^ These hexapeptides correspond to GA sites.


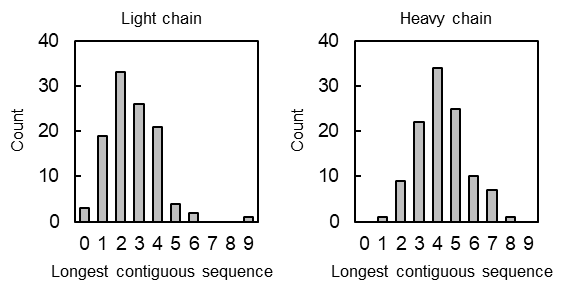


**Figure S1. Length distribution of antigen-binding contiguous sequences in various antibodies.**

The longest contiguous sequences that interact with antigen molecules in light (left) and heavy (right) chains were counted. The binding information of 109 antibody–antigen complexes was collected by a previous study^1^.

**
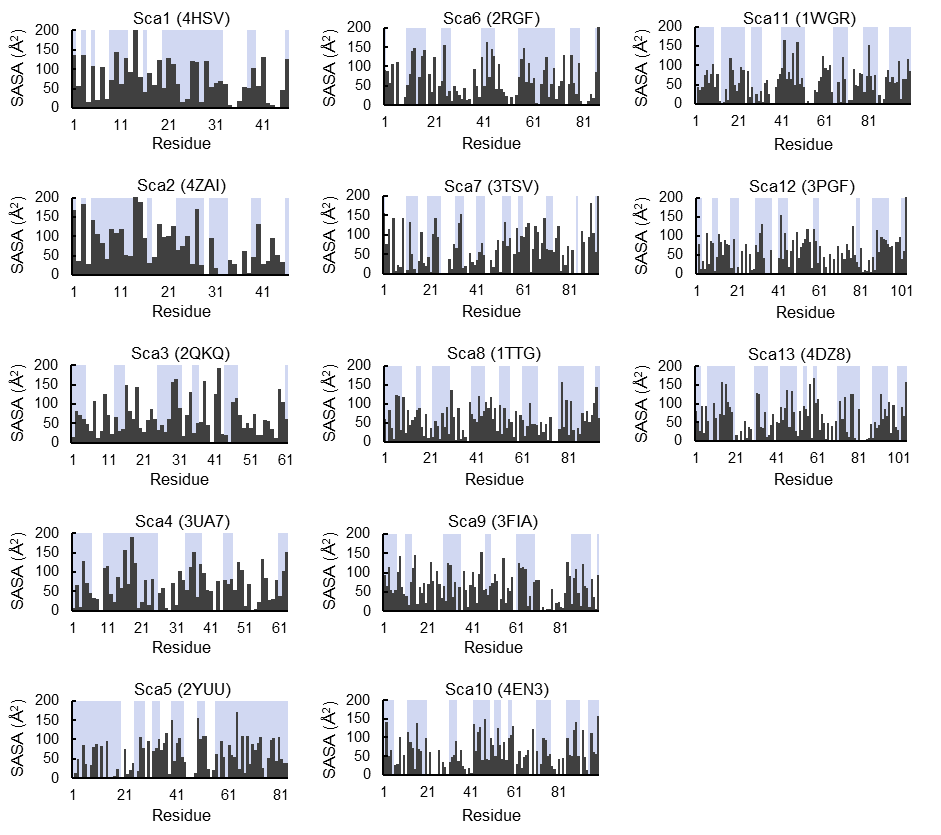
**

**Figure S2. SASA calculation of scaffolds.**

The SASA values of 13 scaffolds were calculated and are shown by residue. The loop regions are highlighted by light blue.


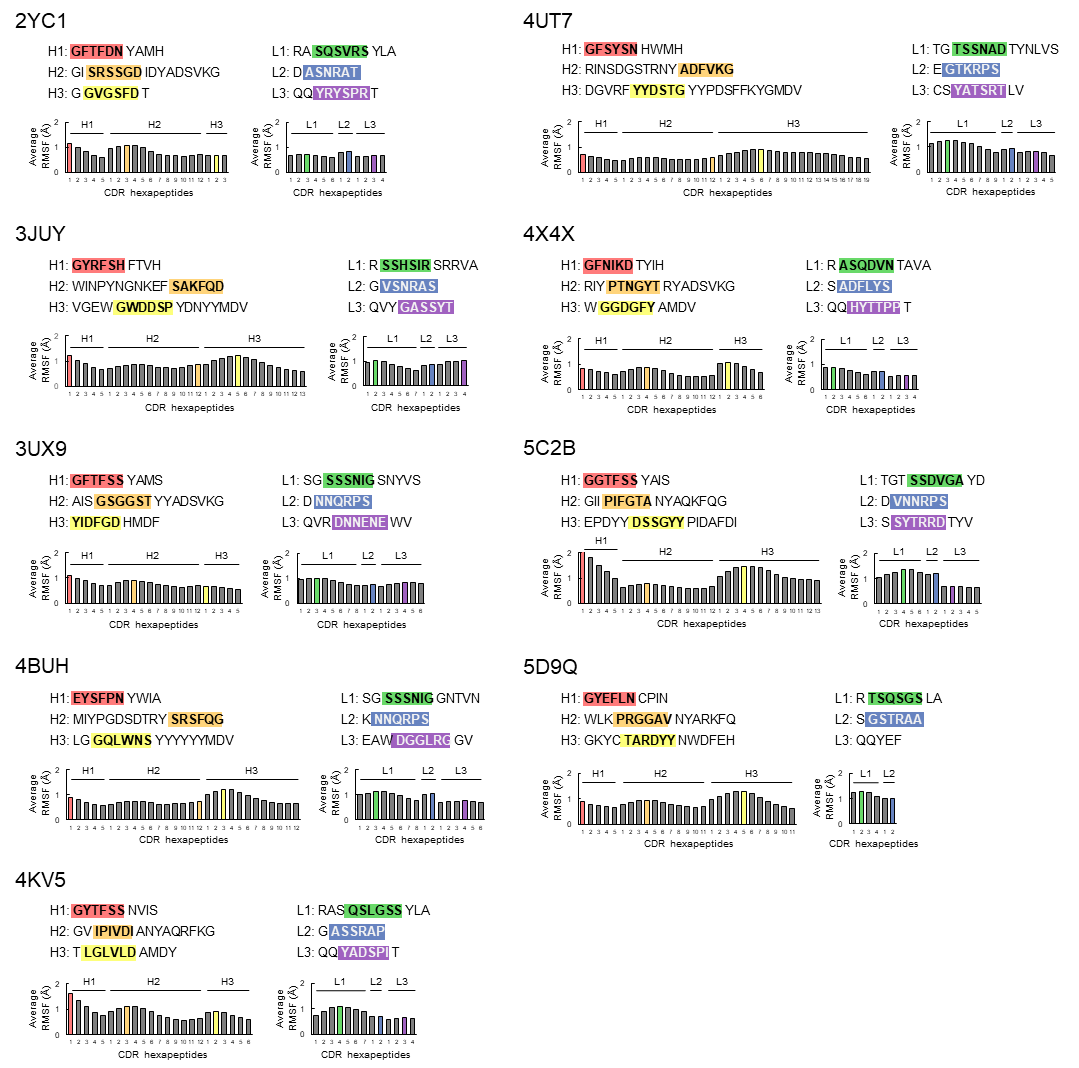


**Figure S3. Structural fluctuation of CDRs in antibodies.**

Nine scFv fragment antibodies indicated by PDB ID were analyzed to investigate the atomic fluctuation of CDR hexapeptides. Amino acid sequences of six CDR loops (H1, H2, H3, L1, L2, and L3) are shown at the top of each panel. The average RMSF values of CDR-derived hexapeptides in scFv structures were calculated and are shown in the graphs under the corresponding amino acid sequences. The most flexible hexapeptides in CDR-H1, -H2, -H3, -L1, -L2, and -L3 (Flex-CDR hexapeptides) are highlighted by red, orange, yellow, green, blue, and purple, respectively. Since CDR-L3 of the 5D9Q antibody is only five amino acids in length, the average RMSF values for this peptide are not shown.


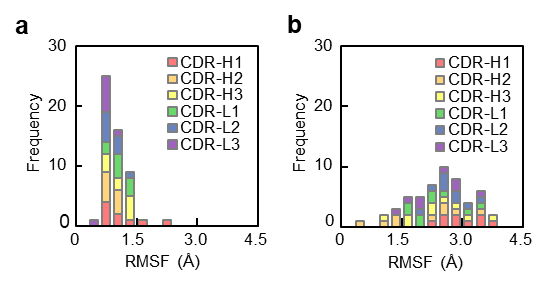


**Figure S4. Peptide-immobilising effect of an antibody.**

(a) Fluctuation of CDR peptides in antibody scFv. The average RMSF values of 53 CDR hexapeptides in nine scFv structures from PDB are shown in the graph. (b) Fluctuation of CDR-derived linear peptides. The average RMSF values of 53 CDR-derived linear peptides that possess the same amino acid sequences as the peptides in (a) are shown in the graph.


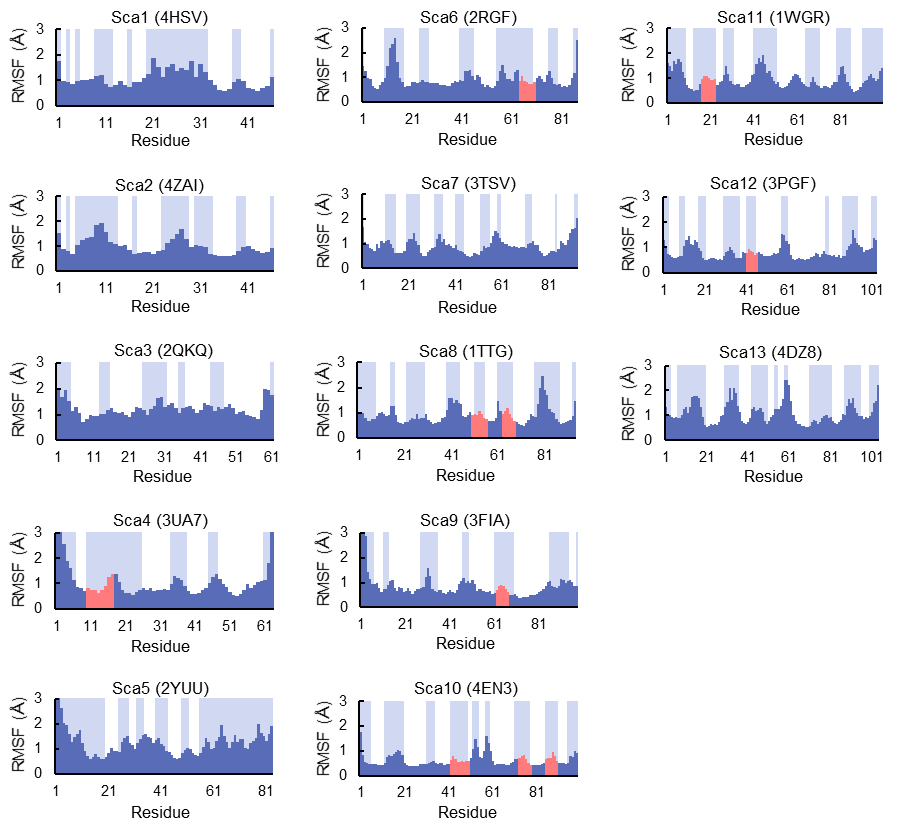


**Figure S5. RMSF calculation of scaffolds.**

The RMSF values of 13 scaffolds were calculated and are shown by residue. The amino acid residues for 18 CSA hexapeptides are shown by pink-coloured bars. Loop regions are highlighted in light blue.


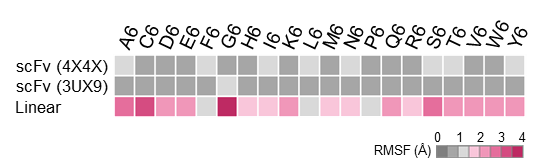


**Figure S6. RMSF profiling of antibodies.**

RMSF profiling of Flex-CDR hexapeptides in scFv scaffolds and linear hexapeptides. Two CDR-H3 loops of scFv proteins (PDB ID: 4X4X and 3UX9) and one computationally-generated linear hexapeptide were profiled. Hexapeptides with average RMSF values of less or more than 1.5 Å are shown by grey- or magenta-coloured squares, respectively.


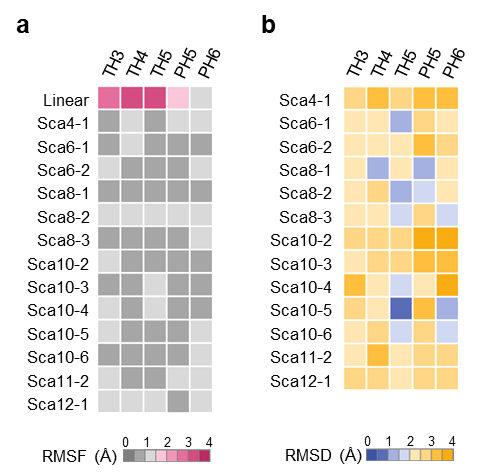


**Figure S7. Fluctuation and shape of anti-HER2 FLAP candidates.**

(a) Structural fluctuation of CDR hexapeptides grafted into scaffolds. The FLAP candidates with an average RMSF value of less or more than 1.5 Å are shown by grey- or magenta-coloured squares, respectively. (b) Structural similarity of CDR hexapeptides grafted into scaffolds with corresponding hexapeptides in antibody-HER2 complex structures. RMSD values of less or more than 2.0 Å are shown by blue- or orange-coloured squares, respectively.


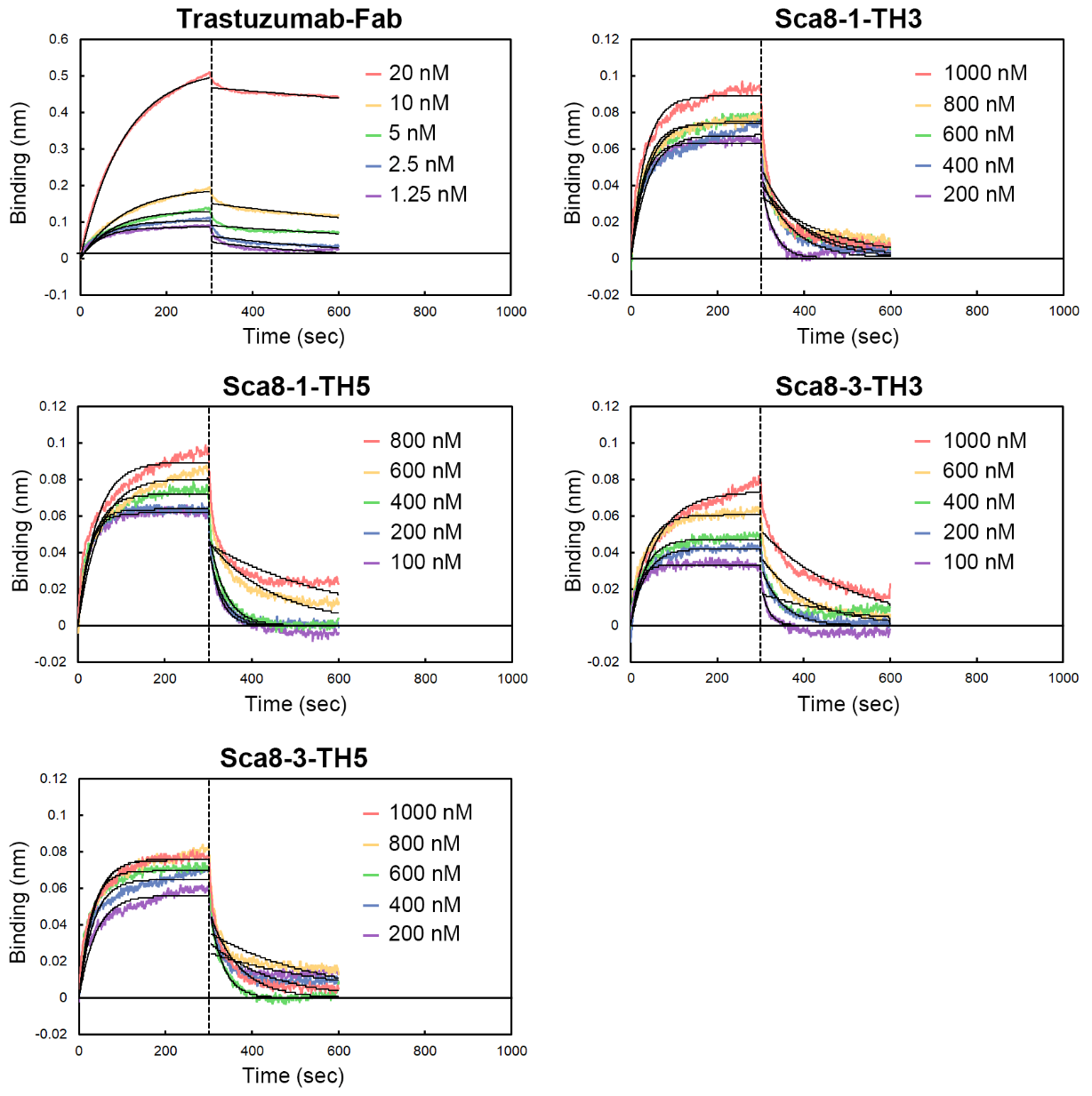


**Figure S8. Affinity measurement by biolayer interferometry.**

Biotinylated HER2-Fc was immobilised on streptavidin biosensors and exposed to various concentrations of trastuzumab-Fab or FLAP candidates. The representative sensorgrams from three experiments were shown.


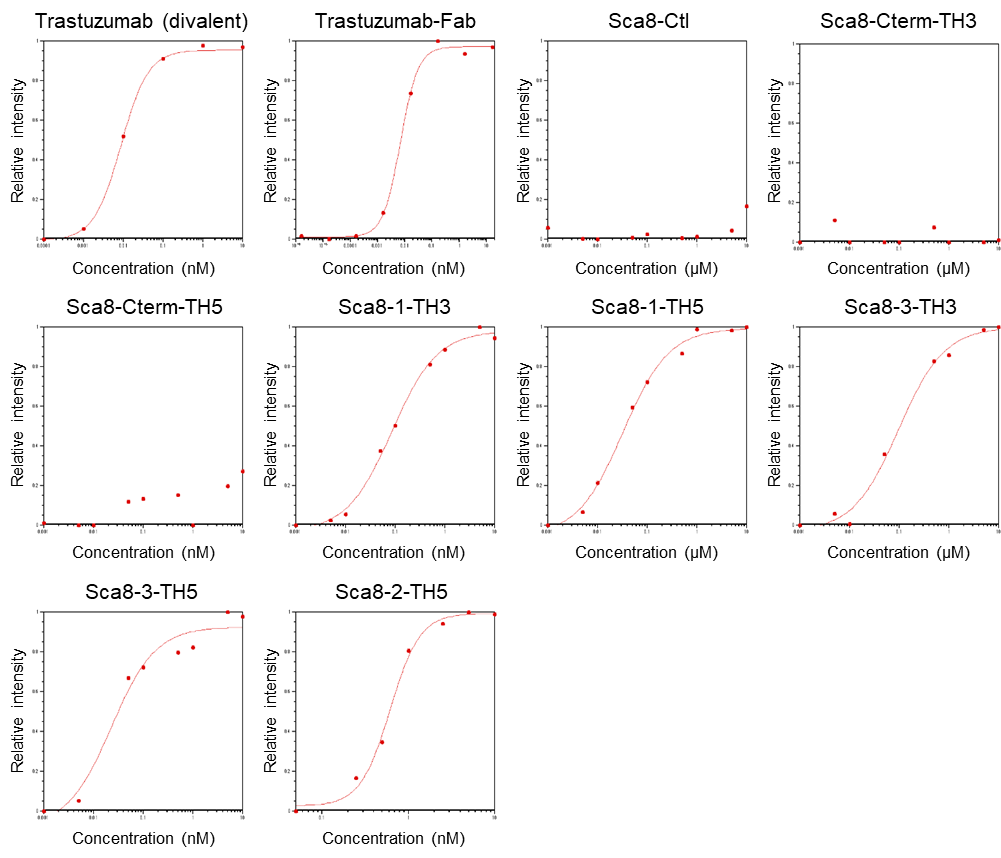


**Figure S9. Affinity measurement by ELISA.**

HER2-Fc was immobilised on ELISA plates and exposed to various concentrations of trastuzumab, trastuzumab-Fab, Sca8-Ctl, or FLAP candidates. Binding was detected with an anti-human Kappa light chain-HRP antibody for trastuzumab, an anti-human IgG (Fab)-HRP antibody for trastuzumab-Fab, or an anti-His tag-HRP antibody for Sca8 and FLAP candidates. The results are representative of three experiments.


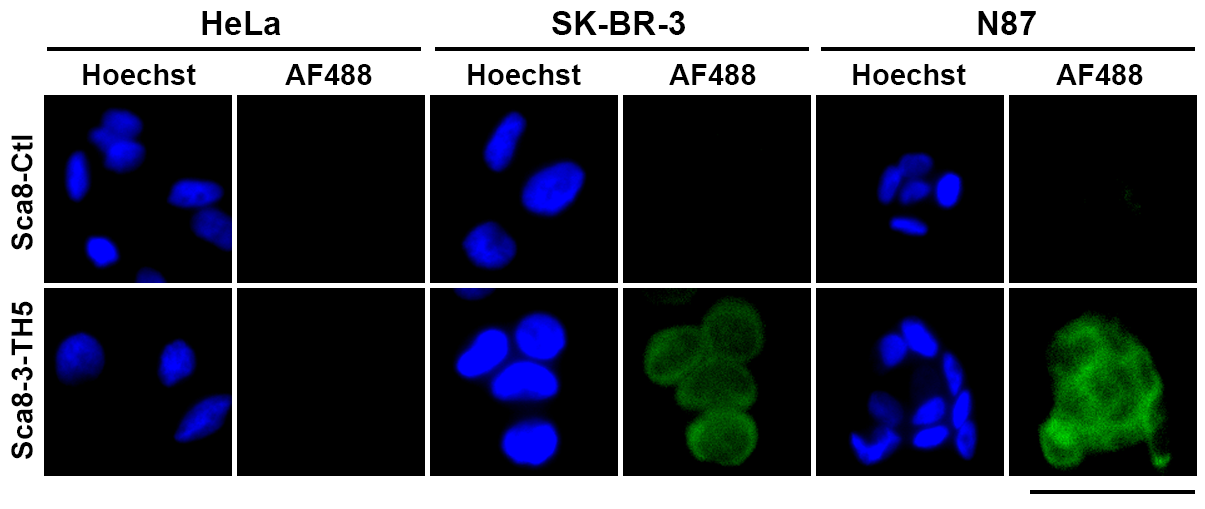


**Figure S10. Immunostaining of HER2-expressing cells with Sca8-3-TH5.**

HeLa, SK-BR-3/Luc, or N87 cells were treated with Sca8-Ctl or Sca8-3-TH5 (green) and Hoechst (blue). Scale bar = 50 µm.

**References**

1. Stave, J. W. & Lindpaintner, K. Antibody and antigen contact residues define epitope and paratope size and structure. *J Immunol* **191(3)**, 1428-1435 (2013).
